# Chemical Characterization and Antioxidant Activity of Nine *Hypericum* Species from Greece

**DOI:** 10.3390/antiox12040899

**Published:** 2023-04-08

**Authors:** Eleni Kakouri, Panayiotis Trigas, Dimitra Daferera, Efstathia Skotti, Petros A. Tarantilis, Charalabos Kanakis

**Affiliations:** 1 Laboratory of Chemistry, Department of Food Science and Human Nutrition, School of Food and Nutritional Sciences, Agricultural University of Athens, Iera Odos 75, 118 55 Athens, Greece; 2 Laboratory of Systematic Botany, Department of Crop Science, School of Plant Sciences, Agricultural University of Athens, Iera Odos 75, 118 55 Athens, Greece; 3 Department of Food Science and Technology, Ionian University, Terma Leoforou Vergoti, 281 00 Argostoli, Cephalonia, Greece

**Keywords:** acylphloroglucinols, antioxidant activity, *Hypericum*, naphthodianthrones, phenolic compounds, phytochemical analysis

## Abstract

*Hypericum* L. comprises about 500 species distributed almost worldwide. Research has mainly focused on *H. perforatum* with confirmed biological activity on the alleviation of depression symptoms, among others. The compounds responsible for such activity are considered naphthodianthrones and acylphloroglucinols. Other *Hypericum* species are less studied or not studied, and further research is needed to complete the characterization of the genus. In this study we evaluated the qualitative and quantitative phytochemical profile of nine *Hypericum* species native to Greece, namely *H. perforatum, H. tetrapterum, H. perfoliatum, H. rumeliacum* subsp. *apollinis, H. vesiculosum, H. cycladicum, H. fragile, H. olympicum* and *H. delphicum*. Qualitative analysis was performed using the LC/Q-TOF/HRMS technique, while quantitative data were calculated with the single point external standard method. Additionally, we estimated the antioxidant activity of the extracts using DPPH and ABTS assays. Three species endemic to Greece (*H. cycladicum, H. fragile, H. delphicum*) were studied for the first time. Our results indicated that all studied species are rich in secondary metabolites, mainly of the flavonoids family, with strong antioxidant activity.

## 1. Introduction

*Hypericum* L. (Hypericaceae) includes about 500 species placed into 36 sections, and has an almost worldwide distribution in temperate, subtropical and mountainous tropical regions [1,2]. Mostly, *Hypericum* spp. are adaptive to mild climate; however, they can also grow in humid or hot climates as well [2]. The Mediterranean Basin is an important diversity center of the genus, hosting more than 150 species distributed into 22 sections [3].

Several *Hypericum* species are known for their medicinal properties and are used in traditional medicine; *Hypericum perforatum* (St. John’s wort), however, is the most widely known species. It has been considered a medicinal plant since ancient times as it has been used for its wound healing and anti-inflammatory properties, and for the relief of gastrointestinal and anxiety disorders, as reported by the Committee on Herbal Medicinal Products [4]. The phytochemical profile of this species has been elucidated by several studies and the presence of various classes of secondary metabolites with interesting pharmacological activity has been discussed [5,6,7,8,9]. The chemical compounds for which the plant has gained popularity as a medicinal agent are the naphthodianthrones (hypericin and pseudohypericin) and the acylphloroglucinol derivates (hyperforin and adhyperforin). Hypericin and pseudohypericin are anthraquinone derivates, products of protohypericin and protopseudohypericin which are formed under the influence of light [10]. According to Robson (1981) [11], hypericin and pseudohypericin are accumulated in the dark glands of leaves and flowers and their presence is typical for the members of sect. *Hypericum*. Both compounds are produced in a higher quantity respective to the other secondary metabolites, and their production is strictly related to environmental conditions [12,13]. However, apart from hypericins, the acylphloroglucinol hyperforin has been evaluated for its pharmacological activity and it has been reported that it is the compound mainly responsible for the antidepressant and anxiolytic properties of *H. perforatum* [14]. Since acylphloroglucinols are found predominantly in the aerial part of the plant, namely fruits and flowers, their production is expected to be higher when fruits are mature enough [15]. Nevertheless, apart from naphthodianthrones and acylphloroglucinols, other classes of compounds identified in *H. perforatum* include cinnamic acids, biflavones, proanthocyanidins, xanthones and subclasses of flavonoids such as flavonols and catechins [16,17].

Naturally derived products are complex mixtures of different chemical compounds, each of which may differently influence biological activity. To this end, when the biological activity of an extract is discussed, usually the phenomenon of pharmacodynamic or pharmacokinetic synergism (or antagonism as well) should be taken under consideration. Consequently, despite the increased scientific interest in naphtnodianthrones and acylphloroglucinols, other classes of compounds such as flavonoids, xanthones, tannins and bioflavonoids, identified at *Hypericum perforatum* extracts, should also be evaluated [18,19].

In contrast to *H. perforatum*, other species of the genus are either less studied or not studied. For instance, only seven out of the twenty-eight members of *H*. sect. *Taeniocarpium*, distributed in the eastern Mediterranean region, have been phytochemically studied to some degree [3]. The lack of phytochemical data increases as the distribution ranges of the species decrease, while only few locally restricted *Hypericum* spp. have been investigated so far, e.g., [20,21].

Therefore, the aim of the present study is to identify the chemical composition of nine *Hypericum* species growing in Greece, namely *H. perforatum* and *H. tetrapterum* (sect. *Hypericum*), *H. perfoliatum, H. rumeliacum* subsp. *apollinis*, *H. vesiculosum* and *H. cycladicum* (sect. *Drosocarpium*), *H. fragile* (sect. *Taeniocarpium*), *H. olympicum* (sect. *Olympia*), and *H. delphicum* (sect. *Adenosepalum*), using liquid chromatography combined with quadrupole time-of-flight high-resolution mass spectrometry (LC/Q-TOF/HRMS), and to evaluate their antioxidant activity with DPPH and ABTS assays. Only limited phytochemical studies have previously been conducted for most of these species, while the phytochemical composition of the Greek endemics *H. delphicum, H. fragile* and the recently described *H. cycladicum* [22] is presented for the first time. Although *H. perforatum* is a well-studied species [6], we included it in the analyses for comparison purposes. Data gathered at this study not only elucidate the phytochemical profile of less studied species, but also demonstrate the potential use of other *Hypericum* species as sources of biologically active compounds.

## 2. Materials and Methods

### 2.1. Chemicals and Reagents

2,2-Diphenyl-1-picrylhydrazyl (DPPH), 2,2′-azinobis-(3-ethylbenzthiazoline-6-sulfonic acid (ABTS) were purchased from Sigma Aldrich (Schnelldorf, Germany). Methanol (LC-MS grade) was purchased from Chem-Lab (Zedelgem, Belgium). Formic acid (LC-MS grade) was purchased from Fischer Scientific (Madrid, Spain); Trolox was purchased from Acrὸs organics (Morris, NJ, USA). Water (LC/MS grade) was purified using a Millipore Direct-Q 3UV apparatus (18.2 mΩ*sec). Standard compounds (catechin, procyanidin B1, epicatechin, chlorogenic acid, myricetin glucoside, kaempferol glucoside, quercitrin, quercetin 3.4-di-O-glucoside, quercetin, kaempferol, rutin, amentoflavone, hypericin) were purchased from ExtraSynthese (Genay, France) and hyperforin was purchased from ChromaDex (Los Angeles, CA, USA).

### 2.2. Plant Material

Plant samples from the nine *Hypericum* species were collected from natural populations in Greece. All samples were collected from full flowering individuals and were dried at 20–25 °C in the dark for further analyses. Information regarding the collected plant material is given in Supplementary Materials, Table S1. Plant material was kept in a dark, dry place for 15 days until use.

### 2.3. Preparation of Extracts

Dried parts (leaves, flowers and stems) of each *Hypericum* species (2 g) were extracted with 30 mL of a methanolic solution (70% *v*/*v*) using an ultrasonic water bath for 15 min under stable temperature (25 ± 1 °C). Extraction was repeated three times. The experiment took place in the dark to avoid degradation of hypericins and hyperforin. The extract was then transferred to a rotary evaporator until complete evaporation of the organic solvent. The final aqueous extract was cooled for 24 h and lyophilized. The obtained powder was kept at −20 °C until further use.

Percentage yield of the dried extracts was calculated using Equation (1):

% yield = [(weight of dry extract)/(weight of dry material)] × 100 (1)

### 2.4. Antioxidant Activity

(a) DPPH free radical antioxidant activity assay

The antioxidant capacity of each sample was measured using the DPPH reagent in methanol according to Kakouri et al. [23]. Different concentrations for each extract were prepared and their absorbance was measured at 517 nm after 30 min of incubation in the dark. Percentage of radical scavenging activity was calculated using Equation (2). Results were expressed as IC_50_ values according to the equation derived from each extract’s plot.

% radical scavenging activity = [(A_0_ − As)/A_0_] × 100 (2)

A standard curve was constructed using Trolox in various concentrations. IC_50_ value was also calculated as for the extracts, for comparison reasons.

(b) ABTS free radical antioxidant activity assay

The antioxidant activity of the extracts was also evaluated using the ABTS assay as previously reported by Kakouri et al. [23]. Absorbance of different concentrations of the samples was measured at 734 nm after six minutes of incubation in the dark. Results were expressed as IC_50_ values, calculated as for the DPPH assay. Trolox was used to construct a standard curve.

### 2.5. Dilution of Standard Compounds

Standard compounds were prepared the day of the experiment in methanol LC-MS. Hypericin was first diluted to the minimum amount of DMSO required and then methanol LC-MS was added.

### 2.6. LC/Q-TOF/HRMS Conditions

Chromatographic analysis was performed on an HPLC 1200 series consisting of a degasser, quaternary pump, autosampler, diode array detector and column oven. The system is coupled to a 6530 Q-TOF mass spectrometer. Analysis was performed on an EC nucleoshell Bluebird RP18, 2.7 μm, 100 × 4.6 mm column at 30 °C. The mobile phase consisted of water acidified with 0.1% (*v*/*v*) formic acid (solvent A) and methanol LCMS grade, acidified with 0.1% (*v*/*v*) formic acid (solvent B). The elution program was operated as follows: 25% solvent B for 0.5 min followed by 10–90% solvent B (0.5–15 min) and 15–25% solvent B (16–21 min). Chromatograms were recorded at 280, 300, 350, 550 and 590 nm; the injection volume was 10 μL and the flow rate was adjusted to 0.74 mL/min. Extracts and standard solutions were prepared the day of the analysis.

The Q-TOF mass spectrometer was operated at the ESI(+) and ESI(−) ionization mode and the parameters set following those of our previous analysis [23]. The mass range (*m*/*z*) was set to 50–1700.

CID MS/MS spectra were recorded on the auto MS/MS mode. The *m*/*z* range was set to 50–800 and collision energy at 40 V. The fragmentor voltage was set at 170 V.

Data were handled using the Agilent MassHunter Workstation software LC-MS Data Acquisition for 6530 series Q-TOF.

Extracts were examined for the presence of secondary metabolites, including phenolic compounds, acylphloroglucinols and naphthodianthrones. Identification was based on available standard solutions (catechin, procyanidin B1, epicatechin, chlorogenic acid, myricetin glucoside, kaempferol glucoside, quercitrin, quercetin 3.4-di-O-glucoside, quercetin, kaempferol, rutin, amentoflavone, hypericin, hyperforin) and the *m*/*z* value of the observed protonated and deprotonated molecular ions. In addition, the “Find compounds by molecular feature” option of the MassHunter software, which generates the molecular formula of the detected compounds, was used. Extracts were analyzed at the ESI(+) and ESI(−) ionization mode, since acylphloroglucinols can form both positive and negative ions, while naphthodianthrones form only negative ions [24].

### 2.7. Quantification of the Identified Compounds: Single Point External Standard Method

The single point external standard method, described by Kanakis et al. [25], was used to quantify the identified compounds. Standard solutions of known concentration were analyzed as described in the LC/Q-TOF/HRMS analysis paragraph. Response factor, i.e., the ratio of the signal produced by the standard solution under a known concentration, was calculated according to the following Formula (3a):

Response factor = (peak area of the standard solution)/(concentration of the standard solution)(3a)

The peak area of the analytes was also recorded and the amount of each analyte was calculated using Equation (3b).

Amount of analyte = (peak area of the analyte)/(response factor)(3b)

Quantification was implemented based on the ESI negative ionization mode. Only for two compounds, namely kaempferol rhamnoside and apigenin hexoside, results were obtained via the ESI positive ionization mode. For compounds where a standard solution was not available, quantification was performed based on standard compounds bearing similar molecular structure. Results were expressed as mg (of the analyte)/g of dry material.

## 3. Results

The present study investigated the qualitative and quantitative phytochemical profile and the antioxidant activity of nine *Hypericum* species collected in Greece. Extraction yield for each species was calculated as the percentage of weight of dry extract divided by the weight of dry starting material. *H. cycladicum* yielded the highest amount, e.g., 26.45% *w*/*w*, followed by *H. perforatum* (23.05% *w*/*w*) and *H. rumeliacum* subsp. apollinis (22.5% *w*/*w*). The % yield of *H. perfoliatum* and *H. delphicum* was close (21 and 21.2% *w*/*w*, respectively), while also close was that of *H. tetrapterum* and *H. fragile* (20.84 and 20.05% *w*/*w*, respectively). *H. vesiculosum* yielded a minor amount equal to 14.2% *w*/*w* and *H. olympicum* yielded the lowest amount (13.85% *w*/*w*).

**Figure 1:**
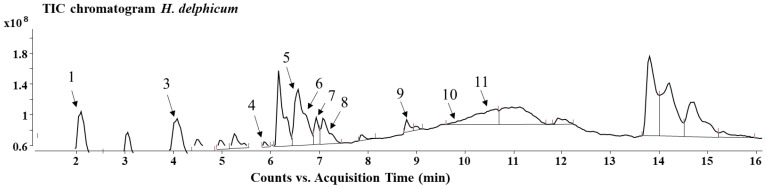
TIC chromatogram of *H. delphicum* extract. **1**: Neo-chlorogenic acid; **2**: chlorogenic acid; **3**: epicatechin; **4** myricetin glucoside; **5**: rutin; **6**: hyperoside; **7**: isoquercitrin; **8**: quercitrin **9**: I3, II8 biapigenin; **10**: amentoflavone; **11**: pseudohypericin.

**Figure 2:**
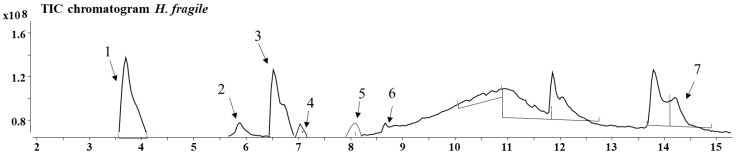
TIC chromatogram of *H. fragile* extract. **1**: chlorogenic acid; **2**: quercetin 3.4-di-O-glucoside; **3**: rutin; **4**: kaempferol rutinoside; **5**: quercetin; **6**: I3, II8 biapigenin; **7**: hyperforin.

**Figure 3:**
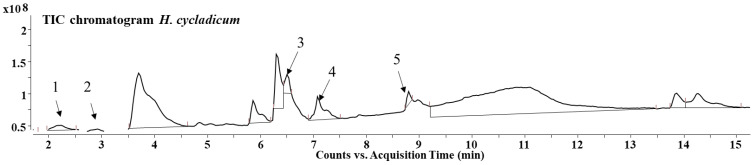
TIC chromatogram of *H. cycladicum* extract. **1**: neo-chlorogenic acid; **2**: procyanidin B-type; **3**: rutin; **4:** kaempferol glucoside; **5**: I3, II8 biapigenin.

Results of the phytochemical analysis of each species are given in Table 1. Detailed information regarding the *m*/*z* experimental versus *m*/*z* theoretical value, mass error in ppm (Δm) and *m*/*z* fragments of the observed ions are given in Supplementary Materials, Tables S2–S10. Total Ion Chromatograms (TIC), at ESI(−), of the newly discussed species, namely *H. cycladicum, H. fragile* and *H. delphicum*, are presented in Figure 1, Figure 2 and Figure 3. In Figures S7–S9 (Supplementary Materials) are given their mass spectra. For the rest of the species, TIC chromatograms are given in Supplementary Materials (Figures S1–S6). In total, 43 compounds were detected mostly belonging to the large family of phenolic compounds. In addition to phenolic compounds, naphthodianthrones, acylphloroglucinols and benzoic acid derivates were also identified. Rutin, a glucoside of quercetin, was a compound identified in all the tested extracts, while the B-type procyanidins, bearing the molecular formulas C_30_H_26_O_14_ and C_45_H_38_O_18_ (procyanidin C1), were only found in the *H. perfoliatum* extract. Myricitrin and p-coumaroyl quinic acid were found only in the extract of *H. rumeliacum* subsp. apollinis; in addition, miquelianin, a quercetin glucuronide, was found only in the extract of *H. perforatum*. On the other hand, *H. delphicum* and *H. perfoliatum* were those samples in which kaempferol rhamnoside was detected. The bi-flavonoid I3, II8 biapigenin was found in all the tested extracts, while amentoflavone was not detected only in two members of *H*. sect. *Drosocarpium*. Finally, none of the quinic acid derivates was detected in the members of *H*. sect. *Hypericum* and sect. *Adenosepalum* tested. In qualitative terms, quinic acid derivates were most abundant in the members of sect. *Drosocarpium*. Hyperforin was detected in the extracts of *H. perfoliatum, H. perforatum* and *H. fragile*, while its homologue adhyperforin was detected only in the extract of *H. perforatum*. Protohypericin was found in both extracts of *H. rumeliacum* subsp. *apollinis* and *H. vesiculosum*. On the other hand, hypericin was found only in the extracts of *H. perforatum* and *H. delphicum*, while pseudohypericin, an oxidized derivative of hypericin, was detected in all extracts except for *H. tetrapterum* (Table 1).

Results of the quantitative analysis were expressed as mg of dry extract/g of dry material. As it can be seen in Table 1, the presence of hyperforin in the *H. perforatum* extract is remarkably high (608.39 mg/g dry material), and the amount of its homologue (adhyperforin) is not negligible (35.53 mg/g dry material). On the contrary, the amount of hyperforin in *H. perfoliatum* and *H. fragile* extracts was very low. As far as naphthodianthrones, the amount of hypericin was approximately threefold higher in the *H. perforatum* extract (7.18 mg/m dry material), respective to that of *H. delphicum* (2.34 mg/g dry material); however, this extract was found to be richer in pseudohypericin (20.87 mg/g dry material). Regarding the family of flavonoids, bi-flavonoids, both I3, II8 biapigenin and amentoflavone, were found in considerable amounts, with I3, II8 biapigenin dominance observed in all the tested extracts. For flavan-3-ols, catechin content ranges from 0.65 to 2.33 mg/g dry material, while for epicatechin the range was found to be between 3.83 and 10.02 mg/g dry material. For all proanthocyanidins identified, a small quantity was calculated. On the contrary, flavones such as luteolin glucoside, myricetin glucoside, myricitrin and flavonols, including kaempferol glucoside and kaempferol rutinoside, were more abundant. Last but not least, chlorogenic acid was found in abundance in *H. rumeliacum* subsp. *apollinis* (46.22 mg/g dry material), *H. cycladicum* (50.13 mg/g dry material) and *H. fragile* (41.6 mg/g dry material) extracts.

Results of the antioxidant activity evaluated by the DPPH and ABTS methods are presented in Table 2. Results are expressed as IC_50_ values. The range of the concentrations used to calculate the DPPH and ABTS free radical scavenging activity of the extracts was the same for all samples. For the DPPH assay this was from 4.95–34.65 μg/mL and for the ABTS assay it ranged from 0.99–9.90 μg/mL (final concentrations). The lowest IC_50_ value corresponds to *H. perforatum* and equals 10.45 ± 0.61 μg/mL for the DPPH assay. *H. vesiculosum* showed the weakest antioxidant activity among the tested samples, with an IC_50_ value equal to 28 ± 1.41 μg/mL. On the other hand, for the ABTS assay, *H. vesiculosum* did not reach an IC_50_ value, while the highest antioxidant activity was estimated in *H. olympicum* extract (IC_50_ = 3.92 ± 0.73 μg/mL).

## 4. Discussion

Among the species of this study, *Hypericum perforatum* is the most studied regarding its phytochemical profile and biological activity. It has been considered an important source of biologically active compounds, and commercially available products standardized in hypericin are currently available. Extracts of the plant are used worldwide to alleviate symptoms of depression as well as to combat major depression of mild to moderate severity, though controversial results of clinical trials question such an effect [26,27,28,29]. However, the majority of the research implemented has established its medicinal use and *H. perforatum* is listed in the European Pharmacopoeia ed. 10.0, as well as in the international pharmacopoeia published by WHO [30,31]. The phytochemical profile of the plant is characterized by the presence of different compounds. The most abundant are kaempferol and quercetin glucosides. Nevertheless, the best-known compounds, which are also considered to be markers of the plant, are the hypericins and hyperforin [32,33]. However, the genus *Hypericum* includes ca. 500 species distributed on all continents except Antarctica, and numerous *Hypericum* spp. remain unexploited. Furthermore, the old origin of the genus dating back at least to Early Cenozoic [34] and its radiation in highly diverse environments raises questions regarding differences in the phytochemical profile among species and therefore their biological activity. As the role of each compound in the biological activity of the extract is not completely resolved [33], in this study, a simple and rapid LC method was used to evaluate the phytochemical profile of the nine *Hypericum* species collected in Greece. This work focused on elucidating the phytochemical profile of the majority of the secondary metabolites that are present in each species, rather than targeting only the family of hypericins and acylphloroglucinols. The gradient program used allowed us to identify flavonoid compounds as well as hypericin and hyperforin, including their precursors and derivatives, respectively. Hyperforin is an unstable compound, strongly susceptible to oxidation under the influence of light and air [25]. Its main oxidation product is furohyperforin [35]. On the contrary, hypericins, although more stable compounds, are also degraded by the influence of light [25].

An approach of the distribution of biomarker compounds was performed by Crockett and Robson, 2011 [2]. They reported that the members of the sections *Hypericum*, *Drosocarpium*, *Taeniocarpium* and *Adenosepalum* are those in which all compounds used for chemotaxonomic classification are present. Some other studies focus on hypericins and report that those are characteristic for the plants of the sections *Hypericum*, *Drosocarpium*, *Oligostema*, *Taeniocarpium* and *Adenosepalum* [36]. On the other hand, hyperforin and its derivates are common compounds of species belonging to the sections *Hypericum* and *Ascyreia* [37]. In addition, acylphloroglucinols have also been identified in sections *Adenosepalum* and *Olympicum*. [38,39]. Hypericin and pseudohypericin are two compounds with the same origin, since they both derive from protohypericin and protopseudohypericin. Exposure to light, acidic or basic pH and storage conditions of their solutions are factors that influence negatively the stability of both compounds [40]. Pseudohypericin, which is usually found in *Hypericum* species at a higher amount than that of hypericin, was detected in all the studied extracts except for *H. tetrapterum*. Regarding *H. tetrapterum*, our results are not in accordance with those of Kitanov et al. (2001), who reported the presence of hypericin and pseudohypericin [41]. However, both pseudohypericin and protohypericin were identified in *H. rumeliacum* subsp. *apollinis* and *H. vesiculosum* of sect. *Drosocarpium*. Hyperforin was detected in the extracts of *H. perfoliatum*, *H. perforatum* and *H. fragile*, and adhyperforin, an acylphloroglucinol with an additional methyl group respective to hyperforin, was only found in the *H. perforatum* extract, which is in accordance with previously reported data [42,43,44].

Apart from the importance of the presence of naphthodianthrones and acylphloroglucinols, the detection of other compounds with significant biological activity has been examined by some researchers [16,45,46]. Napoli et al. (2018) [16] examined some species that belong to different sections for their phytochemistry. Species that belong to the same sections as in our study, namely sect. *Taeniocarpium*, *Adenosepalum*, *Drosocarpium* and *Hypericum*, do present high qualitative similarities. However, the main flavonoids presented in [16] were quercetin glucosides. No rutin or kaempferol (aglycon or glycosides) were detected. Moreover, cinnamic acids were not found in our samples, while no quinic acid derivates were observed in the samples of [16]. Regarding sect. *Taeniocarpium*, *H. fragile* has not yet been studied for its phytochemicals; however, our results on the presence of naphthodianthrones, acylphloroglucinols and some flavonoid compounds are close to those of Camas et al. (2014) [46], who studied, among others, *H. confertum*, *H. linarioides*, *H. pruinatum* and *H. thymifolium* belonging to sect. *Taeniocarpium*.

Regarding *H. perfoliatum*, a less studied species than *H. perforatum*, our results partly agree with those of Del-Monte et al. (2015) [47]. We did not identify caffeic acid(s); on the contrary, a profile more abundant in secondary metabolites was detected. Nedialkov et al. (2007) [45] mention that myricetin and its glucosides are compounds with chemotaxonomic significance for the members of sect. *Drosocarpium*. Although myricetin is a common flavonoid compound presented in many plant extracts, in our study myricetin glucosides were detected in the extracts of *H. rumeliacum* subsp. *apollinis*, *H. vesiculosum*, *H. perforatum H. olympicum* and *H. delphicum*. In addition, myricitrin, a myricetin glucoside, was only found in the *H. rumeliacum* subsp. *apollinis* extract.

*Hypericum cycladicum* (*H*. sect. *Drosocarpium*) is a recently described species endemic to the Cyclades Island group (Aegean Sea) [22], closely related to *H. trichocaulon* and *H. perfoliatum*. No data regarding its chemical composition are available. Therefore, the study of Daskalaki et al. (2021) [21] concerning *H. trichocaulon* was used for comparison purposes. Some common compounds such as chlorogenic acid, I3, II8 biapigenin, hyperoside, quercitrin and rutin were identified in both extracts. However, *H. cycladicum* seems to be richer, in qualitative terms, in secondary metabolites than *H. trichocaulon*. B-type procyanidins, luteolin and kaempferol glucosides and the bi-flavonoid amentoflavone were additionally detected in *H. cycladicum*.

Quantitative and qualitative analysis of the extracts showed differences, not only in the number of secondary metabolites, but also in their concentrations. Generally, it is not always the case that species belonging in the same section are similar regarding their quantitative profile. In our study, the most abundant compounds in all the extracts are flavonoids (except for the extract of *H. perforatum*, in which hyperforin dominates). For the rest of the examined extracts, the flavonoid content of *H. rumeliacum* subsp. *apollinis* reaches 771.41 mg/g dry material. In this extract, the most abundant flavonoids (concentration > 100 mg/g dry material) were myricitrin (304.75 mg/g dry material), kaempferol glucoside (108.93 mg/g dry material) and I3, II8 biapigenin (270.79 mg/g dry material). Napoli et al. (2018) [16] in their study found that species of the sect. *Hypericum* also present quantitative differences. More precisely, *H. perforatum* ethanolic extract was rich in acylphloroglucinols, while for *H. tetrapterum* the flavonoid-type compounds were more abundant. Similarly, for species that belong to sect. *Adenosepalum*, acylphloroglucinols were in abundance (*H. pubescens* extract), while flavonoids prevail in the *H. montanum* extract. Species that belong to the *Drosocarpium* and *Taeniocarpium* sections are also reported to be rich sources of flavonoids. The same applies for *H. vesiculosum* (sect. *Drosocarpium*), in which, according to Zeliou et al. (2020) [48], flavonoids are the main compounds quantified, followed by naphthodianthrones. Our results match with those of [16] and [48]; however, a difference is observed with those of Smelcerovic et al. (2005) [43], regarding hyperoside and quercitrin content. In our study, both hyperoside and quercitrin content were respectively higher in *H. cycladicum* and *H. rumeliacum* subsp. *apollinis* extracts of the sect. *Drosocarpium.*

The importance of antioxidants is an issue intensely discussed due to their multiple health benefits. Their use is not restricted only to the pharmaceutical industry, but antioxidants are also added in several food and cosmetic products [49,50]. The study of the antioxidant activity of plant extracts has become routine in laboratory testing. Many antioxidant assays are used and since differences between them have been observed regarding the obtained results, one extract is most of the time examined via two or more assays. In this work, DPPH and ABTS assays were used to evaluate the antioxidant activity of the samples. *H. perforatum* showed the best antioxidant activity as far as the DPPH assay, followed by *H. delphicum*. For the ABTS assay, all the calculated IC_50_ values were lower than those calculated for the DPPH assay. *H. olympicum* presented an IC_50_ value not far from that of the standard antioxidant, Trolox. The extract of *H. vesiculosum* was the least active for both assays used. Numerous studies on the antioxidant activity of different *Hypericum* species have been published. Taken together, data extracted from these studies make it clear that all *Hypericum* species possess strong antioxidant activity [16,17,51,52,53,54,55]. Our findings match those observed in earlier studies. Certainly, a variation between the estimation of the antioxidant potency is evident; nevertheless, factors including the extraction procedure, the assay used, expression of the results and of course the chemical constituents of an extract are responsible for these differences.

The antioxidant activity of a compound is dependent on its chemical structure [56]. Amentoflavone, a bi-flavonoid detected in the majority of the tested extracts, has strong antioxidant activity, although its structure does not obey the rule of structure–activity and antioxidant capacity [57]. This bi-flavonoid possesses only a double bond at position 2–3 of the C-ring. For the antioxidant activity of I3, II8 biapigenin with a structure similar to amentoflavone, found in abundance in quite all the tested extracts, literature data are not available.

Differences in the antioxidant capacity of the extracts are observed, which is rather expected taking into account the qualitative and quantitative variation between the identified secondary metabolites. All the tested extracts possess a significant antioxidant activity. Five out of nine species contain secondary metabolites of the flavan-3-ols and proanthocyanidins family. Proanthocyanidins are polyphenolic compounds, formed by flavan-3-ols subunits bound with B or A linkages. Most common flavan-3-ols subunits are catechin, gallocatechin and their isomers, from which oligo- or polymeric proanthocyanidins can be formed, among which oligo- proanthocyanidins are reported to be more effective antioxidants [58]. Literature data mention that oligo- proanthocyanidins are more potent as antioxidants than vitamins E and C [59]. In this study, B-type dimers and trimers proanthocyanidins were identified in the extracts of *H. perfoliatum, H. vesiculosum, H. cycladicum, H. perforatum* and *H. delphicum*. However, the *H. cycladicum* extract, apart from the above-mentioned family of biologically active compounds, also contains a great amount of chlorogenic acid, the highest in comparison with the rest of the extracts. Luteolin glucoside and kaempferol glucoside are present. Further, the sum of flavonoids is high. For the ABTS assay, the calculated IC_50_ value of *H. olympicum* extract is the lowest. Despite the sum of flavonoids presented in this extract not being the highest one, some secondary metabolites, in particular myricetin glycosides, are detected in considerable amount. Myricetin is a strong antioxidant compound [56]. Both glycosides were also tentatively identified in the *H. rumeliacum* subsp. *apollinis* extract, although in minor quantity. Myricitrin was also identified in this extract at a significant amount. Also of note, the *H. rumeliacum* subsp. *apollinis* extract is the richest regarding the sum of flavonoids and the second one in abundance of chlorogenic acid. *H. cycladicum* is another species with interesting antioxidant activity. The presence of luteolin glucoside and the higher amount of chlorogenic acid and amentoflavone, respective to the other extracts, contribute to its activity.

Although the number of compounds identified in the extract of *H. vesiculosum* is higher, a better antioxidant activity was estimated in the case of the *H. delphicum* extract. The sum of flavonoids for the two extracts is close; however, the better antioxidant activity for *H. delphicum* might be attributed to myricetin glucoside and kaempferol rhamnoside, which are present at great amounts in this extract. Generally, flavonoids are found in plants mostly in their glycosylated form, in particular at positions 3 (O-glycosylation) and 7 (C-glycosylation). As has been suggested, glycosylated forms are less active in terms of antioxidant activity. In addition, the number and type of glycosidic moieties alter the antioxidant activity, with glucose being the sugar to cause the minimum interference [60]. Most of the compounds detected in our study are flavonoid glycosides of kaempferol and quercetin. The only aglycons identified were catechin, epicatechin and kaempferol in the extracts of *H. perfoliatum*, *H. perforatum*, *H. delphicum* (only epicatechin was identified) and *H. cycladicum* (only kaempferol was identified). Taken together, data of the antioxidant activity highlight that qualitative and quantitative differences are crucial for the radical scavenging activity of an extract. Definitely, a synergistic effect of secondary metabolites, including flavonoids, proanthocyanidins, quinic and benzoic acid derivates, contributes to the overall results. Naphthodianthrones and acylphloroglucinols seem not to contribute to the antioxidant activity [52].

## 5. Conclusions

This study evaluated the phytochemical profile of nine *Hypericum* species native to Greece, among which the endemic species of *H. cycladicum*, *H. fragile* and *H. delphicum* are studied for the first time. Generally, most of the studied species are overlooked compared to *H. perforatum*. Therefore, the present study adds novel information on the phytochemical profile and the antioxidant activity of the genus *Hypericum*.

Different families of secondary metabolites were identified, all documented by the existing literature to have a significant biological activity; hence, the present study gives strong motivation for further research on the biological activity of more species of the genus that will create a new perspective for their use. Our results present some similarities compared to those species for which studies have been performed. Nevertheless, important differences are also mentioned that highlight the unique climate characteristics between the different locations where the plant is found. However, the high demand for herbal products, in combination with exogenous factors such as climate change, pose risks for medicinal plants. As mentioned by [61], most medicinal plants used for industrial purposes are collected in the wild. This poses several questions regarding the equivalence of the final products in terms of standardization, and at the same time raises serious thoughts that need straightforward answers, since wild plant material is not inexhaustible. Therefore, after evaluating the bioactivity of a plant material, the first problem to solve is its collection and cultivation under specific agro-ecological conditions, as well as the cost of such a procedure including the creation of a final commercial product.

## Figures and Tables

**Table 1:** Phytochemical analysis of Hypericum extracts expressed as mg/g of dry material.

		Sect. *Drosocarpium*				Sect. *Hypericum*		Sect. *Taeniocarpium*	Sect. *Olympia*	Sect. *Adenosepalum*
Compound	Molecular Formula	*H. perfoliatum*	*H. rumeliacum* subsp. *apollinis*	*H. vesiculosum*	*H. cycladicum*	*H. perforatum*	*H. tetrapterum*	*H. fragile*	*H. olympicum*	*H. delphicum*
**Flavan-3-ols** **and Proanthocyanidins**										
Procyanidin B type	C_30_H_26_O_14_	0.01	-	-	-	-	-	-	-	-
Catechin *	C_15_H_14_O_6_	0.65	-	-	-	2.33	-	-	-	-
Procyanidin B1 *	C_30_H_26_O_12_	0.61	-	-	1.26	2.06	-	-	-	-
Procyanidin B type	C_30_H_26_O_12_	2.20	-	0.05	1.41	-	-	-	-	-
B-type trimer procyanidin (C1)	C_45_H_38_O_18_	0.42	-	-	-	-	-	-	-	-
B-type trimer procyanidin (C2)	C_45_H_38_O_18_	1.65	-	-	1.93	2.02	-	-	-	2.68
Epicatechin *	C_15_H_14_O_6_	3.83	-	-	-	9.21	-	-	-	10.02
**Quinic acid derivates**										
Neo-chlorogenic acid	C_16_H_18_O_9_	-	13.51	4.94	6.61	-	-	-	15.83	17.49
Chlorogenic acid *	C_16_H_18_O_9_	-	46.22	0.05	50.13	0.20	36.97	41.6	28.56	0.46
p-coumaroylquinic acid	C_16_H_18_O_8_	-	1.04	-	-	-	-	-	-	-
**Benzoic acid derivates**										
Vanillic acid hexoside	C_14_H_8_O_9_	-	-	n.q^1^	-	-	-	-	-	-
**Flavones**										
Apigenin hexoside	C_21_H_20_O_10_	4.73	-	-	3.74	-	-	-	-	-
Luteolin malonylhexoside	C_24_H_22_O_14_	4.03	-	5.82	3.44	-	-	-	-	-
Luteolin glucuronide	C_21_H_18_O_12_	0.13	-	-	-	-	-	-	-	-
Luteolin glucoside *	C_21_H_20_O_11_	-	-	-	32.34	-	-	-	-	-
Myricetin glucoside *	C_21_H_20_O_13_	-	55.89	1.38	-	13.02	-	-	315.52	68.98
Myricetin arabinoside	C_20_H1_8_O_12_	-	24.78	-	-	-		-	64.99	-
Myricitrin *	C_21_H_20_O_12_	-	304.75	-	-	-	-	-	-	-
**Flavonols**										
Hyperoside	C_21_H_20_O_12_	0.011	0.02	1.17	3.59	-	-	0.008	1.48	0.01
Isoquercitrin	C_21_H_20_O_12_	1.39	0.02	0.01	3.98	4.26	1.67	1.13	0.01	2.55
Kaempferol glucoside *	C_21_H_20_O_11_	6.60	108.93	-	33.24	-	-	-	-	-
Quercitrin *	C_21_H_20_O_11_	0.011	4.94	0.36	1.51	-	-	-	-	0.90
Kaempferol malonylhexoside	C_24_H_22_O_14_	0.88	-	1.61	1.03	-	-	-	-	-
Miquelianin	C_21_H_18_O_13_	-	-	-	-	0.65	-	-	-	-
Quercetin arabinofuranoside	C_20_H_18_O_11_	-	0.93	-	-	-	-	-	-	2.78
Quercetin 3.4-di-O-glucoside *		-	-	-	-	-	0.06	3.61	-	-
Quercetin glucoside acetate	C_23_H_22_O_13_	-	-	-	-	0.28	-	-	-	-
Quercetin malonylhexoside	C_24_H_22_O_15_	0.14	-	0.23	0.15	-	-	-	-	-
Kaempferol rutinoside	C_27_H_30_O_15_	-	-	11.39	4.70	-	2.63	5.30	-	-
Quercetin *	C_15_H_10_O_7_	-	0.06	0.25	-	0.31	-	0.11	015	-
Kaempferol *	C_15_H_10_O_6_	-	-	-	0.003	-	-	-	-	-
Kaempferol rhamnoside	C_21_H_20_O_10_	-	-	-	-	4.92	-	-	-	10.64
Rutin *	C_27_H_30_O_16_	0.13	0.30	3.89	0.14	0.13	0.24	22.21	0.18	0.55
**Bi-flavonoids**										
I3.II8 biapigenin	C_30_H_18_O_10_	4.18	270.79	181.11	371.12	176.92	130.75	54.73	48.73	158.90
Amentoflavone *	C_30_H_18_O_10_	-	-	23.95	102.94	14.39	11.83	9.44	6.23	19.54
**Naphthodianthrones**										
Pseudohypericin	C_30_H_16_O_9_	0.99	5.44	1.40	8.52	0.007	-	4.57	0.10	20.87
Hypericin *	C_30_H_16_O_8_	-	-	-	-	7.18	-	-	-	2.34
Protohypericin	C_30_H_18_O_8_	-	2.65	0.31	-	-	-	-	-	-
**Acylphloroglucinols**										
Hyperforin *	C_35_H_52_O_4_	0.38	-	-	-	608.39	-	0.05	-	-
Adhyperforin	C_36_H_54_O_4_	-	-	-	-	35.53	-	-	-	-

* Identification based on standard compounds.

**Table 2:** Antioxidant activity of *Hypericum* spp. extracts by DPPH and ABTS assays.

Species	DPPH Assay	ABTS Assay
	IC_50_ (μg/mL) *	IC_50_ (μg/mL) *
*H. perfoliatum*	13.32 ± 2.03	4.71 ± 1.63
*H. rumeliacum* subsp. *apollinis*	16.14 ± 0.68	5.07 ± 107
*H. vesiculosum*	28.39 ± 1.41	-
*H. cycladicum*	16.8 ± 1.02	6.80 ± 1.46
*H. perforatum*	10.45 ± 0.61	6.06 ± 0.93
*H. tetrapterum*	17.85 ± 1.57	8.64 ± 1.43
*H. fragile*	23.99 ± 1.24	7.86 ± 0.91
*H. olympicum*	14.1 ± 0.94	3.92 ± 0.73
*H. delphicum*	12.98 ± 1.09	5.84 ± 1.28
Trolox	4.84 ± 1.15	2.84 ± 0.54

* Data are mean +/− standard error of the mean (SEM), *n* = 3.

## Data Availability

Data is contained within the article and supplementary material.

## Supplementary Materials

The following supporting information can be downloaded at: https://www.mdpi.com/article/10.3390/antiox12040899/s1, Tables S1–S10. Table S1. Collection data of the *Hypericum* species examined; Table S2: Tentatively identified compounds in *H. perfoliatum* extract; Table S3: Tentatively identified compounds in *H. rumeliacum* subsp. *apollinis* extract; Table S4: Tentatively identified compounds in *H. vesiculosum* extract; Table S5: Tentatively identified compounds in *H. cycladicum* extract; Table S6: Tentatively identified compounds in *H. perforatum* extract; Table S7: Tentatively identified compounds in *H. tetrapterum* extract; Table S8: Tentatively identified compounds in *H. fragile* extract; Table S9: Tentatively identified compounds in *H. olympicum* extract; Table S10: Tentatively identified compounds in *H. delphicum* extract. Figures S1–S9. Figure S1: TIC chromatogram of *H. perfoliatum* extract; Figure S2: TIC chromatogram of *H. rumeliacum* subsp. *apollinis* extract; Figure S3: TIC chromatogram of *H. vesiculosum* extract; Figure S4: TIC chromatogram of *H. perforatum* extract; Figure S5: TIC chromatogram of *H. tetrapterum* extract; Figure S6: TIC chromatogram of *H. olympicum* extract; Figure S7: Mass spectrum of *H. delphicum* extract; Figure S8: Mass spectrum of *H. cycladicum* extract; Figure S9: Mass spectra of *H. fragile* extract.
