# Supplementary material for: Chemical Characterization and Antioxidant Activity of Nine Hypericum Species from Greece
Source: Antioxidants (Basel). 2023 Apr 8;12(4):899. doi: 10.3390/antiox12040899 (PMC10135362; doi:10.3390/antiox12040899)
Supplement: Supplementary file 1 [file antioxidants-12-00899-s001.zip › antioxidants-2156301-supplementary.pdf]

Chemical characterization and antioxidant activity of nine *Hypericum* species from Greece

Eleni Kakouri[1], Panayiotis Trigas[2], Dimitra Daferera[1], Efstathia Skotti[3], Petros A. Tarantilis[1], Charalabos Kanakis[1]*

[1] Laboratory of Chemistry, Department of Food Science and Human Nutrition, School of Food and Nutritional Sciences, Agricultural University of Athens, Iera Odos 75, 118 55, Greece

[2] Laboratory of Systematic Botany, Department of Crop Science, School of Plant Sciences, Agricultural University of Athens, Iera Odos 75, 118 55, Greece

[3] Department of Food Science and Technology, Ionian University, Terma Leoforou Vergoti, 28100 Argostoli, Cephalonia, Greece

*Correspondence: chkanakis@aua.gr; Tel.: +30-2105294249

Table S1. Collection data of the *Hypericum* species examined

Taxon	Collection site	Latitude	Longitude	Elevation (m)	Habitat	Voucher number
H. cycladicum	Andros Island	37º54'	24º52'	30	Phrygana	12285
H. delphicum	Evvia Island	38º53'	23º52'	1000	Forest clearings	12281
H. fragile	Evvia Island	38º32'	24º01'	420	Cliffs	12283
H. olympicum	Evvia Island	38º36'	23º51'	890	Rocky slopes	12288
H. perfoliatum	Mt. Parnon	37º15'	22º39'	1050	Forest	12282
H. perforatum	Andros Island	37º50'	24º53'	560	Rocky slopes	12280
H. rumeliacum subsp. *apollinis*	Mt. Parnassos	38º33'	22º34'	1760	Rocky slopes	12286
H. tetrapterum	Evvia Island	38º36'	23º51'	910	Wet places	12287
H. vesiculosum	Mt. Chelmos	38º05'	22º10'	910	Woodland	12284

Table S2: Tentatively identified compounds in *H. perfoliatum* extract

Hypericum perfoliatum									
**Compound name**	**Rt**	**ESI+**	**ESI-**	***m/z* experimental**		***m/z* theoretical**		***Δm***	
		**Product ions**	**Product ions**	**+**	**-**	**+**	**-**	**+**	**-**
Procyanidin B type	1.39	307.9972;275.9969; 263.0556; 139.0344; 127.0365	-	611.1398	-	611.1395		0.44	
B-type trimer procyanidin (C1)	1.58	-	739.1455;714.14; 695.1275;451.09; 289.0708;245.04; 179.0442	-	865.2014	-	865.1985	-	3.35
Procyanidin B1	2.28	-	standard solution	577.1331		577.1351			-3.46
Catechin	2.80	standard solution	standard solution	291.0854	289.0717	291.0863	289.0717	-3.12	0
Procyanidin B type	3.03	425.1754;291.0852; 287.0536	407.0761;289.070; 245.0811;205.0503	579.1477	577.134	579.1497	577.1351	-3.45	-1.9
B-type trimer procyanidin (C2)	3.69	-	713.1518;695.1398; 577.1325;451.1012; 289.0710;243.0298; 161.0243; 125.024	-	865.1966	-	865.1985	-	-2.2
Epicatechin	3.89	standard solution	standard solution	291.0851	291.0863 1	289.0716	289.0717	-4.12	-0.34
Apigenin hexoside	5.76	271.0581; 153.017; 121.0275; 119.05	-	433.1114	-	433.1129	-	-3.46	-
Luteolin malonylhexoside	6.14	287.0526; 137.0231; 135.0408	285.0394;257.0448; 241.0499;133.0294;	535.1067	533.0923	535.1082	533.0937	-2.80	-2.63
Rutin	6.53	-	Standard solution	-	609.1449	-	609.1461	-1.99	
Hyperoside	6.55	303.0483; 153.0172	301.0317; 283.0266; 271.0222	465.1012	463.0872	465.1027	463.0882	-3.33	-2.15
Luteolin glucuronide	6.58	-	300.0269;243.0287; 241.0095;217.0453; 119.0402;175.0354; 151.0035	-	461.0717	-	461.0725	-	-1.84

Isoquercitrin	6.73	303.0489	301.0316; 300.0274; 283.0248; 229.0479	465.1009	463.0872	465.1027	463.0882	-3.98	-2.16
Quercetin malonylhexoside	6.79	-	301.0323;283.0243; 229.0477;151.0038; 107.0149	-	549.087	-	549.0885	-	-2.73
Quercitrin	7.04	standard solution	standard solution	449.106	447.0918	449.1078	447.0933	-4.00	-3.35
Kaempferol glucoside	7.21	standard solution	standard solution	449.1081	447.0918	449.1078	447.0933	0.67	-3.35
Kaempferol malonylhexoside	7.50	287.0535; 241.043; 231.0594;258.0471; 165.0188;153.0153; 133.0266; 121.0268	285.0388;267.0287; 243.0283;239.0336; 213.0536;199.0387; 151.0045	535.1065	533.0923	535.1082	533.0937	-3.23	-2.63
I3.II8 biapigenin	8.82	-	443.0428;385.0718; 151.0038	-	537.0808	-	537.0827	-3.57	-3.54
Pseudohypericin	10.11	-	487.0458;457.0784; 459.0951	-	519.0702	-	519.0722	-3.85	-3.77
Hyperforin	14.57	-	standard solution	-	535.377	-	535.3793	-	4.30

Table S3: Tentatively identified compounds in *H. rumeliacum* subsp. *apollinis* extract

***H. rumeliacum* subsp. apollinis**									
**Compound name**	**Rt**	**ESI+**	**ESI-**	***m/z* experimental**		***m/z* theoretical**		***Δm***	
				+	-	+	-	+	-
Neo-chlorogenic acid	2.24	-	191.0558;135.0448; 85.0294	-	353.0871	-	353.08781	-	-2.01
Chlorogenic acid	3.70	standard solution	standard solution	355.1019	353.0875	355.1024	353.0878	-1.41	-0.85
p-Coumaroylquinic acid	5.03	-	191.0558;163.1114; 137.0229; 119.0497	-	337.0923	-	337.0929		-1.75
Myricetin glucoside	5.80	-	standard solution	-	479.0824	-	479.0831	-	-1.46
Myricetin arabinoside	6.26	-	316.0220;299.0169; 271.0237;245.0425; 151.003; 137.0243	-	449.0719	-	449.0725	-	-1.34
Myricitrin	6.3	standard solution	standard solution	465.1023	463.0874	465.1027	463.0882	-0.86	-1.73
Rutin	6.50	-	standard solution	-	609.1437	-	609.14611		-3.94

Hyperoside	6.55	303.0485;153.0175	316.0217; 300.027; 281.0072;271.0243; 229.0216	465.1022	463.0872	465.1027	463.0882	-1.07	-2.16
Isoquercitrin	6.9	-	316.0217;271.0241; 255.0295; 151.0036	-	463.0873	-	463.0882	-	-1.94
Kaempferol glucoside	7.11	-	standard solution	-	447.0924	-	447.0932	-	-1.79
Quercetin arabinofuranoside	7.13	-	300.0273;283.0226; 271.0244;229.0121; 169.0278; 151.0039	-	433.0767	-	433.0776	-	2.08
Quercitrin	7.15	standard solution	standard solution	449.1072	447.0923	449.10784	447.0932	-1.33	-2.01
Quercetin	7.85	-	standard solution	-	301.0347	-	301.03538		-2.32
I3.II8 biapigenin	8.78	445.0501; 387.0839; 153.0173	443.0381;385.0705; 151.0034	539.0969	-	539.09727	-	-0.74	-
Pseudohypericin	10.22	-	519.0698;487.0437; 421.06	-	519.0704	-	519.07216	-	-3.47
Protohypericin	11.32	-	433.0705;407.0827; 363.1039		505.0926		505.0929		-0.59

Table S4: Tentatively identified compounds in *H. vesiculosum* extract

H. vesiculosum									
**Compound name**	**Rt**	**ESI+**	**ESI-**	***m/z* experimental**		***m/z* theoretical**		***Δm***	
				+	-	+	-	+	-
Vanillic acid hexoside	2.03	-	167.0329;152.0107; 137.0218;123.0438; 164.0676	-	329.0873	-	329.0878	-	-1.55
Neo-chlorogenic acid	2.24	-	191.0560;161.0246; 127.0397	-	353.0875	-	353.0878	-	-0.88
Procyanidin B type	3.01	-	407.0747;289.0708; 245.0816; 205.0513	-	577.1349	-	577.1351	-	-0.35
Chlorogenic acid	3.45		standard solution	-	353.0882		353.0878	-	1.13
Myricetin glucoside	5.87	-	standard solution	-	479.081		479.0831	-	-4.38
Luteolin malonylhexoside	6.04	287.0531;137.0227; 135.0433	285.0392;257.0467; 241.049; 175.04;	535.10 84	533,0926	535. 1082	533,0937	0. 37	-2,06

			133.0295						
Rutin	6.52	-	standard solution	-	609.1447	-	609.1461	-	-2.3
Hyperoside	6.53	303.0491; 153.0179	301.0322;300.0269; 283.0257; 271.0244	465.10 23	463,0874	465. 1027	463,0882	- 0. 86	-1,73
Quercetin malonylhexoside	6.81	303.0493;285.0379; 257.0433;229.0472; 165.0187;153.0174; 149.0243; 137.0218	301.0328;300.0269; 283.0229;229.0487; 151.0031;107.0137;	551.10 33	549,0873	551. 1031	549,0885	0. 36	-2,18
Isoquercitrin	6.81	-	301.322; 300.0268; 271.0241;255.0295; 151.0037	-	463.0873	-	463.0882	-	-1.94
Quercitrin	7.05	-	standard solution	-	447.0922	-	447.0932	-	-2.24
Kaempferol rutinoside	7.12	-	285.0392;267.0296; 257.0294;241.0497; 239.036; 213.0551; 199.0402; 151.003	-	593.1500	-	593.1511	-	-1.85
Kaempferol malonylhexoside	7.53	-	285.0387;267.0306; 239.0355;213.0574; 199.0401; 151.0031	-	533.0927	-	533.0937	-	-1.87
Quercetin	7.92	-	standard solution	-	301.0351	-	301.0353	-	-0.66
I3.II8 biapigenin	8.86	-	443.0396;385.0702; 151.0033	-	537.0814	-	537.0827	-	-2.42
Amentoflavone	9.95	-	standard solution	-	537.081	-	537.0827		-3.16
Pseudohypericin	10.03	-	519.07; 487.0421; 471.046	-	519.0701	-	519.0722	-	-4.05
Protohypericin	11.78	-	435.085; 407.074	-	505.0933	-	505.0923	-	-1.98

Table S5: Tentatively identified compounds in *H. cycladicum* extract

H. cycladicum									
**Compound name**	**Rt**	**ESI+**	**ESI-**	***m/z* experimental**		***m/z* theoretical**		***Δm***	
				+	-	+	-	+	-
Neo-chlorogenic acid	2.24	-	191.0566; 135.0449; 85.0299	-	353.0871		353.0878	-	-1.98
Procyanidin B1	2.49	-	standard solution	-	577.1345	-	577.1351	-	-1.04
Procyanidin B type	3.00	-	407.0766; 289.0717; 245.082; 205.0482	-	577.1345	-	577.1351	-	-1.04
Chrologenic acid	3.46	-	standard solution	-	353.0871	-	353.0878	-	-1.98
Procyanidin B type-Procyanidin C2	3.78	-	695.1386;575.1138; 287.0534;243.0275; 161.0243; 125.0243	-	865.1981	-	865.1985	-	-0.42
Apigenin hexoside	5.8	271.0581;153.0167; 121.0283; 119.05	-	433.1121	-	433.1129	-	-1.85	-
Luteolin malonylhexoside	5.99	287.0544;137.0235; 135.0458	285.0383; 257.047; 241.0498; 175.04; 133.0293	535.1073	533.0923	535.1082	533.0937	-1.68	-2.63
Luteolin glucoside	6.33	-	standard solution	-	447.0924	-	447.0933	-	-2.01
Rutin	6.52	-	standard solution	-	609.1448	-	609.1461	-	-2.13
Hyperoside	6.56	303.0491; 153.0168	301.0319;300.0271; 283.0248; 271.0246	465.1019	463.0874	465.1027	463.0882	-1.72	-1.73
Isoquercitrin	6.82	303.0515	301.0319;300.0271; 283.0248; 271.0246	487.084*	463.0874	487.085*	463.0882	-2.05	-1.73
Quercetin malonylhexoside	6.83	303.0488;285.0385; 257.0442;229.0493; 165.0167;153.0175; 149.021; 137.0228	301.0331;300.0271; 229.0188;151.0031; 107.105	551.1025	549.0872	551.1031	549.0885	-1.09	-2.18
Quercitrin	6.98	standard solution	standard solution	449.1067	447.0923	449.1078	447.0933	-2.45	-2.24
Kaempferol glucoside	7.05	-	standard solution	-	447.0924	-	447.0933	-	-2.01
Kaempferol rutinoside	7.13	287.0539;258.0511; 231.0657;213.0491; 165.0152;153.0174; 133.0304; 121.0286	285.0394; 267.029; 255.0296;241.0511; 239.0336; 213.056; 199.0388; 151.0032	595.1645	593.1499	595.1657	593.1511	-2.02	-2.02

Kaempferol malonylhexoside	7.53	-	285.0383;267.0264; 239.0323;211.0382; 199.0412; 151.0023	-	533.0926	-	533.0937	-	-2.06
Kaempferol	8.61	-	standard solution	-	285.0396		285.04	-	-1.4
I3. II8 biapigenin	8.82	445.0533;387.0859; 153.0162; 121.0276	443.0401;385.0703; 151.0033	539.0965	537.0811	539.0973	537.0827	-1.48	-2.98
Amentoflavone	9.95	-	standard solution	-	537.0813	-	537.0827	-	-2.61
Pseudohypericin	10.25	-	519.0704;487.0489; 475.0844	-	519.0703	-	519.0722	-	-3.66

*M+Na

Table S6: Tentatively identified compounds in *H. perforatum* extract

H. perforatum									
**Compound name**	**Rt**	**ESI+**	**ESI-**	***m/z* experimental**		***m/z* theoretical**		***Δm***	
				+	-	+	-	+	-
ProcyanidinB1	2.28	-	standard solution	-	577.1329	-	577.1351	-	-3.81
Catechin	2.78	standard solution	standard solution	291.0856	289.0714	291.0863	289.0717	-2.4	-1.04
Chlorogenic acid	3.46	-	standard solution	-	353.0871	-	353.0878	-	-1.98
Procyanidin B type-Procyanidin C2	3.75	-	695.1375; 452.102; 243.031; 161.0242; 125.0242	-	865.1968	-	865.1985	-	-1.96
Epicatechin	3.99	standard solution	standard solution	291.0853	289.0716	291.0863	289.0717	-3.43	-0.35
Kaempferol rhamnoside	4.52	287.0534; 269.048; 241.0479;213.0532; 259.059; 231.065; 153.0192	–	433.1115	–	433.1129	-	-3.23	-
Myricetin glucoside	5.85	-	standard solution	-	479.0817	-	479.0831	-	-2.92
Rutin	6.49	-	standard solution	-	609.1444	-	609.1461	-	-2.79
Miquelianin	6.71	-	301.0347;283.0252; 273.0389;229.0494; 151.0034; 107.0138	-	477.0663	-	477.0674	-	-2.51
Isoquercitrin	6.88	303.0498	301.0325; 300.0265; 283.0244; 229.0486	487.0833*	463.0869	487.085*	463.0882	-3.49	-2.81

Quercetin glucoside acetate	7.48	-	300.0267;271.024;255.029; 227.0333	-	505.0971	-	505.0988	-	-3.36
Quercetin	7.88	standard solution	standard solution	303.0487	301.035	303.0499	301.0354	-3.96	-1.33
I3. II8 biapigenin	8.84	-	443.0385; 385.0705; 151.0037	-	537.081	-	537.0827	-	-3.16
Amentoflavone	9.95	-	standard solution	-	537.081	-	537.0827	-	-3.16
Pseudohypericin	10.5	-	519.0695;487.0447; 475.0754	-	519.0705	-	519.0722	-	-3.27
Hypericin	12.34	-	standard solution	-	503.0768	-	503.0772	-	-0.79
Hyperforin	14.66	standard solution	standard solution	537.3933	535.3773	537.3938	535.3793	-0.93	-3.73
Adhyperforin	14.99	-	549.3954;480.3215; 411.2521;397.2372; 313.18; 289.1438	-	549.3931	-	549.3949	-	-3.29

*M+Na

Table S7: Tentatively identified compounds in *H. tetrapterum* extract

H. tetrapterum									
**Compound name**	**Rt**	**ESI+**	**ESI-**	***m/z* experimental**		***m/z* theoretical**		***Δm***	
				+	-	+	-	+	-
Chlorogenic Acid			standard solution	-	353.0874	-	353.0878	-	-1.13
Quercetin 3,4-di-O-glucoside	5.7	-	standard solution	-	625.1398	-	625.141	-	-1.92
Rutin	6.46	-	standard solution	-	609.1448	-	609.1461	-	-2.13
Isoquercitrin	6.97	-	301.0333;300.0269; 271.0242;255.0303; 151.0047	-	463.0862	-	463.0882	-	-4.32
Kaempferol rutinoside	7.15	-	285.0393;267.0276; 255.0292;241.0485; 239.0323;213.0537; 199.0404; 151.0027	-	593.1495	-	593.1512	-	-2.87
I3. II8 biapigenin	8.87	-	443.0373;385.0698; 151.0035	-	537.0808	-	537.0827	-	-3.54
Amentoflavone	9.92	-	standard solution	-	537.0809	-	537.0827	-	-3.35

Table S8: Tentatively identified compounds in *H. fragile* extract

H. fragile									
**Compound name**	**Rt**	**ESI+**	**ESI-**	***m/z* experimental**		***m/z* theoretical**		***Δm***	
				+	-	+	-	+	-
Chlorogenic acid	3.65	-	standard solution	-	353.0874	-	353.0878	-	-1.13
Quercetin 3,4-di-O-glucoside	5.7	-	standard solution	-	625.1401	-	625.1410	-	-1.44
Rutin	6.54	standard solution	standard solution	611.1583	609.1447	611.1607	609.1461	-3.86	-2.99
Hyperoside	6.58	303.0478; 155.0177	301.0229;300.01892; 271.0199	465.1012	463.0876	465.1027	463.0882	-3.22	1.29
Isoquercitrin	6.96	-	301.0332;300.0281; 271.024; 255.0301; 151.0036	-	463.0867	-	463.0882	-	-3.24
Kaempferol rutinoside	7.15	-	285.0393;267.0305; 255.0296;241.0499; 239.0337;213.0556; 199.0399; 151.0031	-	593.1496	-	593.1512	-	-2.7
Quercetin	7.87	standard solution	standard solution	303.0484	301.0349	303.0495	301.0354	-3.63	-1.66
I3.II8 biapigenin	8.88	-	443.0384;385.0707; 151.0035	-	537.0808	-	537.0827	-	-3.54
Amentoflavone	9.91	-	standard solution	-	537.0806	-	537.0827	-	-3.91
Pseudohypericin	10.36	-	519.0706; 487.042; 475.0797	-	519.0706	-	519.0722	-	-3.08
Hyperforin	14.56	-	standard solution	-	535.3775	-	535.3793	-	-3.36

Table S9: Tentatively identified compounds in *H. olympicum* extract

H. olympicum									
**Compound name**	**Rt**	**ESI+**	**ESI-**	***m/z* experimental**		***m/z* theoretical**		***Δm***	
				+	-	+	-	+	-
Neo-chlorogenic acid	2.24	-		-	353.0877	-	353.0878	-	-2.26
Chlorogenic acid	3.69		standard solution	-	353.0871	-	353.0878		1.98
Myricetin glucoside	5.85	standard solution	standard solution	481.0953	479.0822	481.0977	479.0832	-4.93	-2.09
Myricetin arabinoside	6.21	319.0423;153.0169; 165.0167	316.0214;299.0162; 271.0239;151.0024;	451.0853	449.0714	451.0871	449.0725	-3.99	-2.45

			137.0252						
Rutin	6.54	-	standard solution	-	609.1449	-	609.1461	-	-1.97
Hyperoside	6.56	303.0477; 285.0384;273.0377; 229.047	316.0225;301.0300; 281.1023;151.0052	465.1007	463.0873	465.1027	463.0882	-4.41	-1.94
Isoquercitrin	6.84	-	300.0268;271.0244; 255.0294; 151.0031	-	463.0868	-	463.0882	-	-3.02
Quercetin	7.86	-	standard solution	-	301.035	-	301.0354	-	-1.33
I3. II8 biapigenin	8.84	-	443.0378;385.0713; 151.0032	-	537.0812	-	537.0827	-	-2.79
Amentoflavone	9.96	-	standard solution	-	537.0814	-	537.0827	-	-2.42
Pseudohypericin	10.35	-	519.0703;487.0437; 421.1124	-	519.0712	-	519.0721	-	-1.73

Table S10: Tentatively identified compounds in *H.delphicum* extract

H. delphicum									
**Compound name**	**Rt**	**ESI+**	**ESI-**	**m/z experimental**		**m/z theoretical**		**Δm**	
				**+**	**-**	**+**	**-**	**+**	**-**
Neo-chlorogenic acid			191.0579; 135.0452; 85.0301		353.0874		353.0878	-	-1.32
Chlorogenic acid	3.45	-	standard solution	-	353.0880	-	353.0878	-	0.56
Procyanidin B type-Procyanidin C2	3.75	-	713.1532;695.1414; 243.0308;161.0241; 125.0239; 577.1331	-	865.1966	-	865.1985	-	-2.2
Epicatechin	4.08	standard solution	standard solution	291.0849	289.0715	291.0863	289.0717	-4.81	-0.69
Kaempferol rhamnoside	4.38	287.053;269.0402;241.0465; 259.0594; 231.064; 241.0465;213.0534;165.0172	-	433.1108	-	433.1129	-	-4.85	-
Myricetin glucoside	5.83	-	standard solution	-	479.0818	-	479.0831	-	-2.71
Rutin	6.57	-	standard solution	-	609.1449	-	609.1461	-	1.97
Hyperoside	6.58	303.0479;153.0169	301.0325;300.0272; 283.0242;271.0245	465.1007	463.0874	465.1027	463.0882	-4.3	-1.73
Isoquercitrin	6.73	303.0481;153.0174	301.0321;300.0270;	465.1006	463.0872	465.1027	463.0882	-4.51	-2.16

			271.0245;255.0295; 151.0035						
Quercitrin	7.15	-	standard solution	-	447.0921	-	447.0933	-	-2.68
Quercetin arabinofuranoside	7.18	-	301.032; 300.0264; 283.0223;271.0244; 151.0033	-	433.0763	-	433.0776	-	-3
I3. II8 biapigenin	8.84	445.0528; 387.084; 153.017	443.0365;385.0709; 151.0035	539.0972	537.081	539.0947	537.0827	-4.51	4.63
Amentoflavone	9.9	-	standard solution	-	537.0813	-	537.0827	-	-2.6
Pseudohypericin	10.36	-	519.0706;487.042; 475.0797	-	519.0706	-	519.0722	-	-3.08
Hypericin	12.44	-	standard solution	-	503.0777	-	503.0772	-	0.99

TIC chromatograms of *Hypericum* species at the negative ionization mode

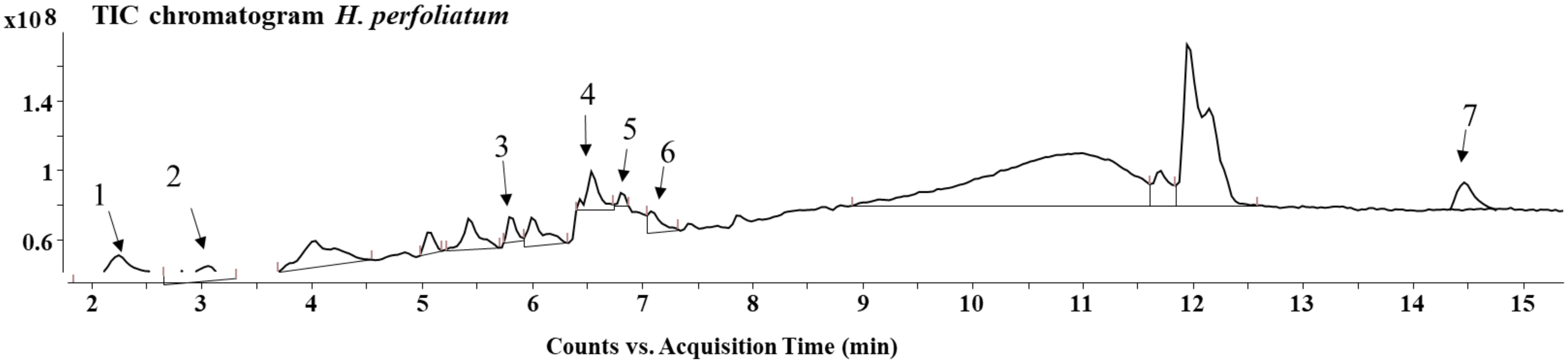

Figure S1: TIC chromatogram of *H. perfoliatum* extract. **1**: procyanidin B1; **2**: procyanidin B type; **3**: apigenin hexoside; **4:** rutin; **5**: quercetin malonylhexoside; **6**: quercitrin; **7**: hyperforin

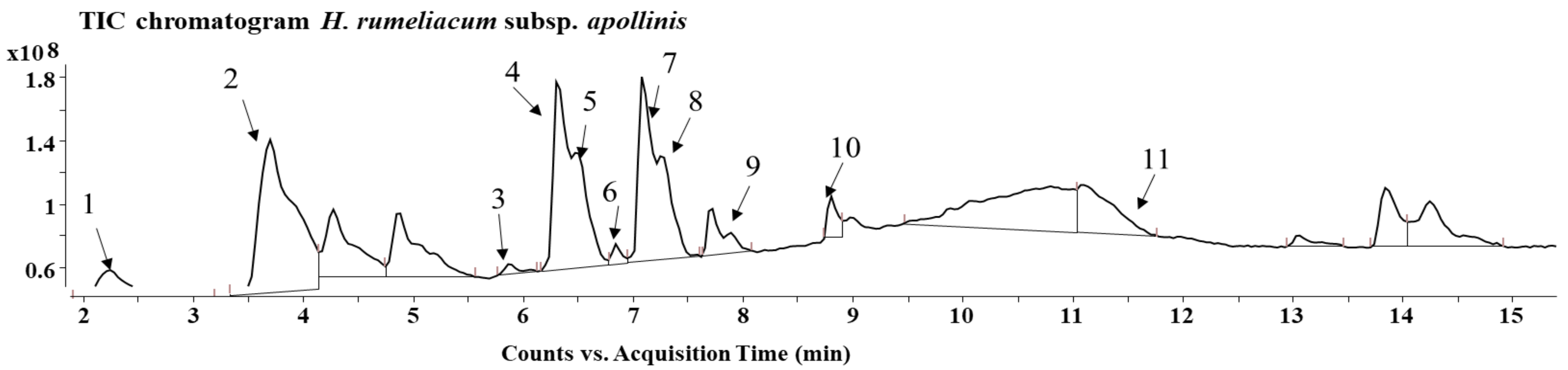

Figure S2: TIC chromatogram of *H. rumeliacum* subsp. *apollinis* extract. **1**: neo-chlorogenic acid; **2**: chlorogenic acid; **3**: myricetin glucoside; **4:** myricitrin; **5**: rutin; **6**: isoquercitrin; **7**: kaempferol glucoside; **8**: quercitrin; **9**: quercetin; **10**: I3, II8 biapigenin; **11**: protohypericin

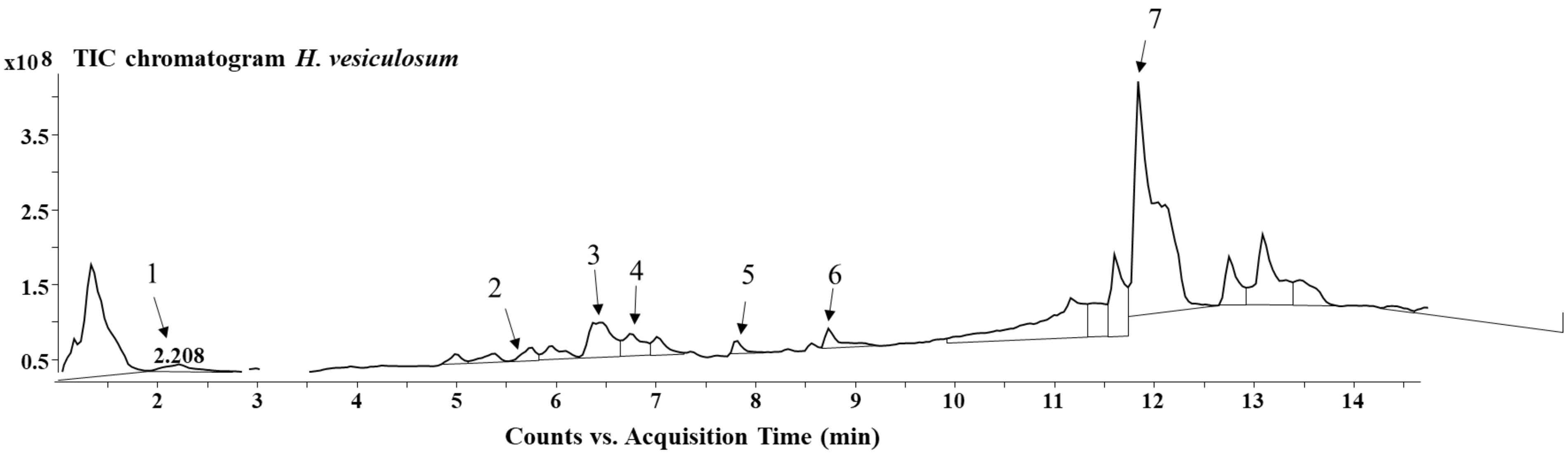

Figure S3: TIC chromatogram of *H. vesiculosum* extract. **1**: neo-chlorogenic acid; **2**: myricetin glucoside; **3**: quercetin malonylhexoside; **4:** isoquercitrin; **5**: quercetin; **6**: I3, II8 biapigenin; **7**: protohypericin

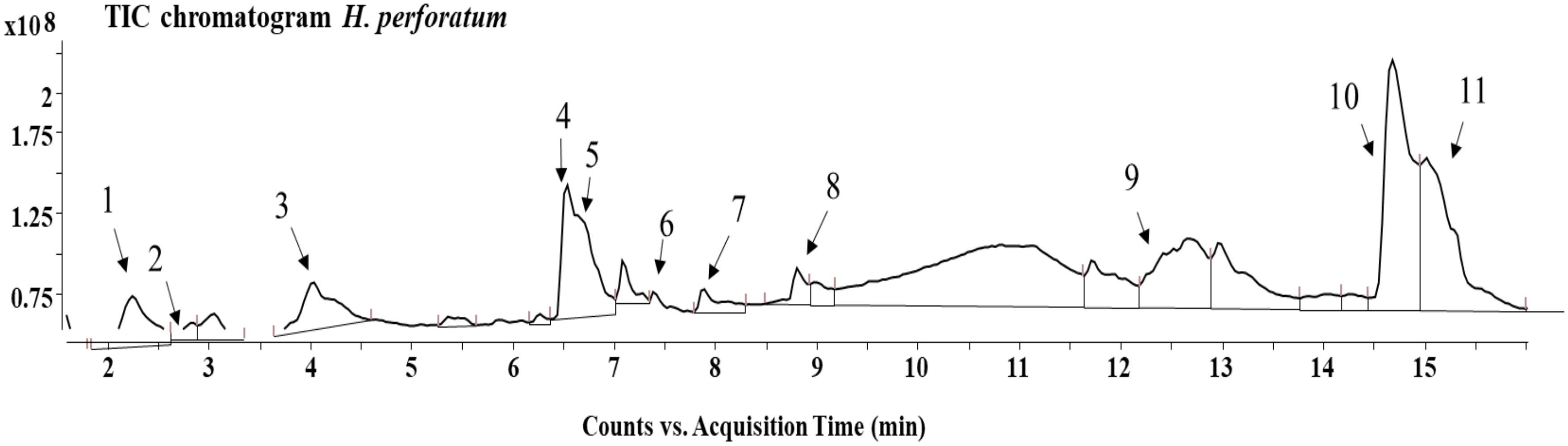

Figure S4: TIC chromatogram of *H. perforatum* extract. **1**: procyanidin B1; **2**: catechin; **3**: epicatechin; **4:** myricetin glucoside; **5**: rutin; **6**: quercetin glucoside acetate; **7**: quercetin; **8**: I3, II8 biapigenin; **9**: hypericin; **10**: hyperforin; **11**: adhyperforin

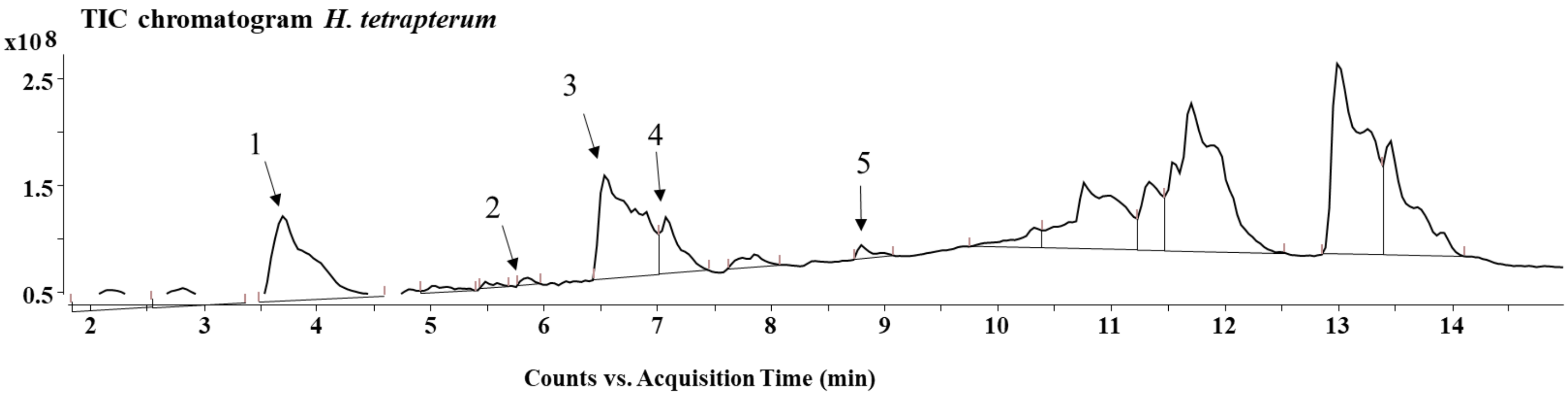

Figure S5: TIC chromatogram of *H. tetrapterum* extract. **1**: chlorogenic acid; **2**: quercetin 3,4-di-O-glucoside; **3**: rutin; **4**: isoquercitrin; **5:** I3, II8 biapigenin glucoside

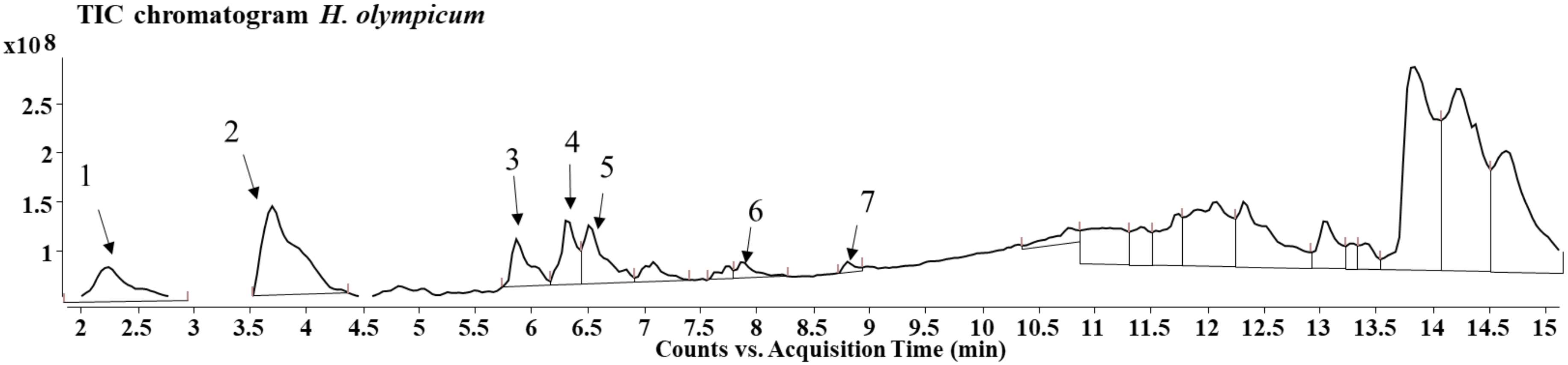

Figure S6: TIC chromatogram of *H. olympicum* extract. 1: Neo-chlorogenic acid; **2**: chlorogenic acid; **3**: myricetin glucoside; **4**: myricetin arabinoside; **5:** rutin; **6**: quercetin; **7**: I3, II8 biapigenin

Figure S7: Mass spectrum of *H. delphicum* extract

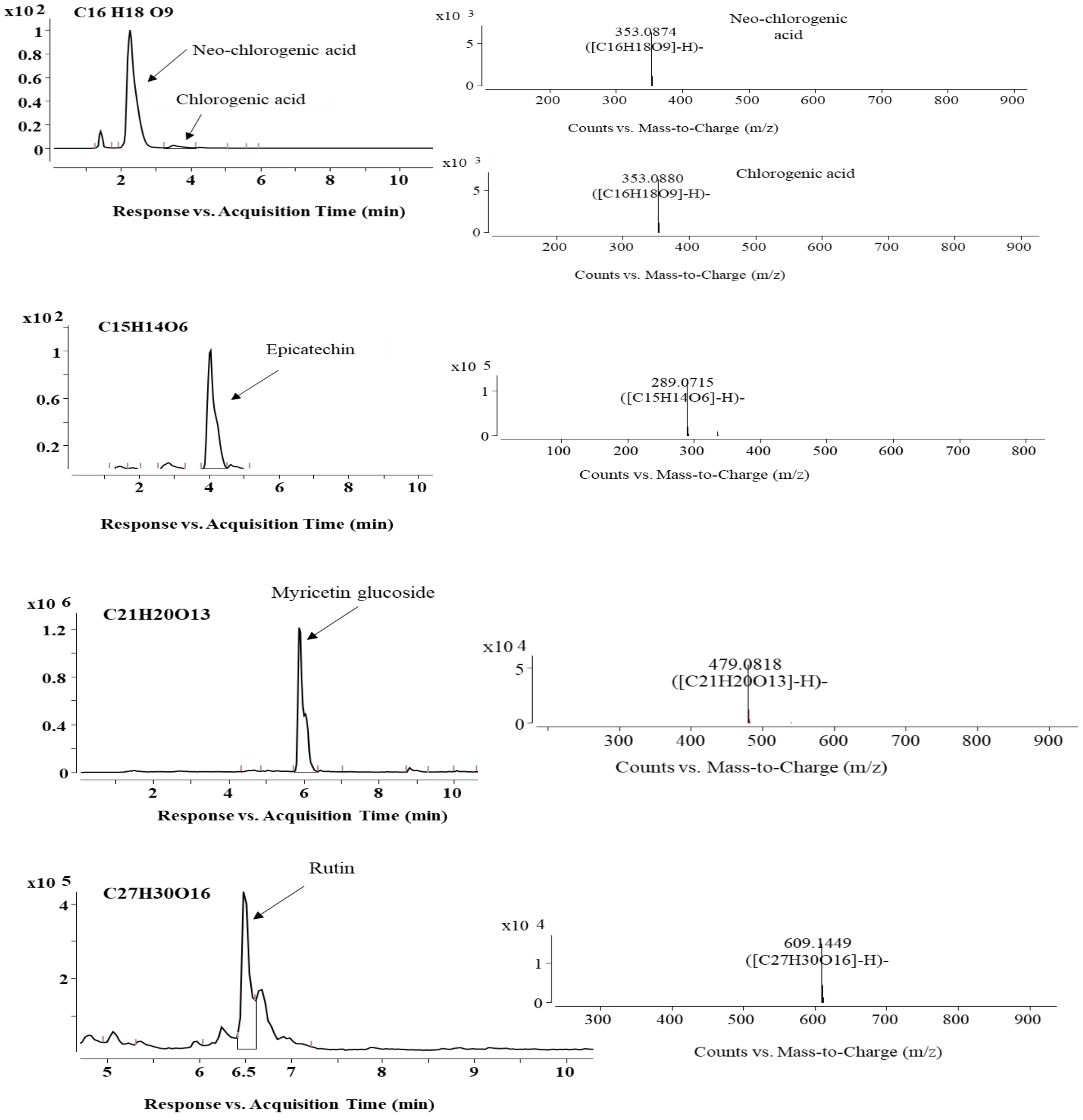

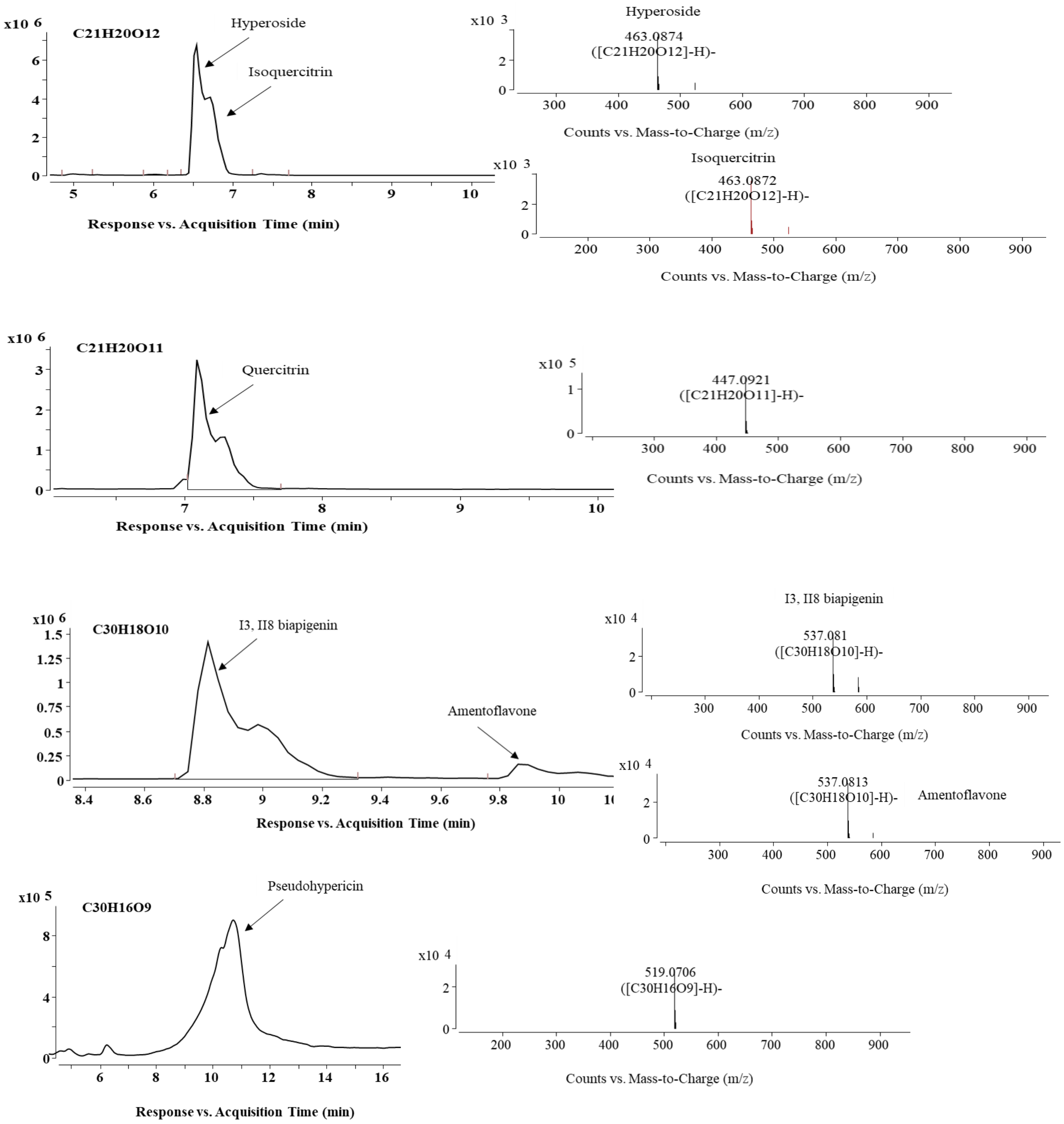
C21H20O12
Hyperoside
Isoquercitrin
Response vs. Acquisition Time (min)
463.0874
([C21H20O12]-H)-
Counts vs. Mass-to-Charge (m/z)
463.0872
([C21H20O12]-H)-
C21H20O11
Quercitrin
447.0921
([C21H20O11]-H)-
C30H18O10
I3, II8 biapigenin
Amentoflavone
537.081
([C30H18O10]-H)-
537.0813
([C30H18O10]-H)-
C30H16O9
Pseudohypericin
519.0706
([C30H16O9]-H)-

Figure S8: Mass spectrum of *H. cycladicum* extract

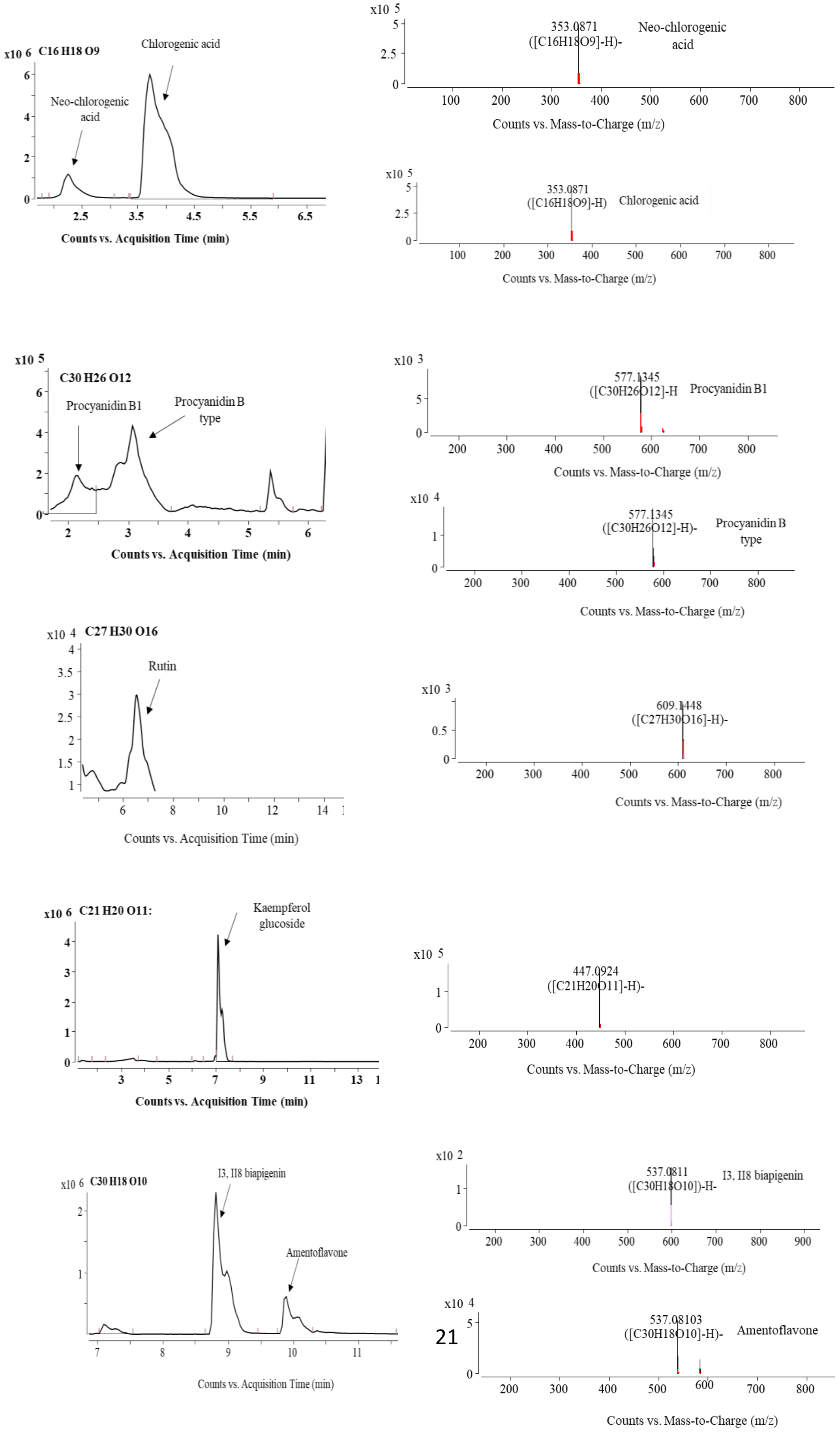

Figure S9: Mass spectra of *H. fragile* extract

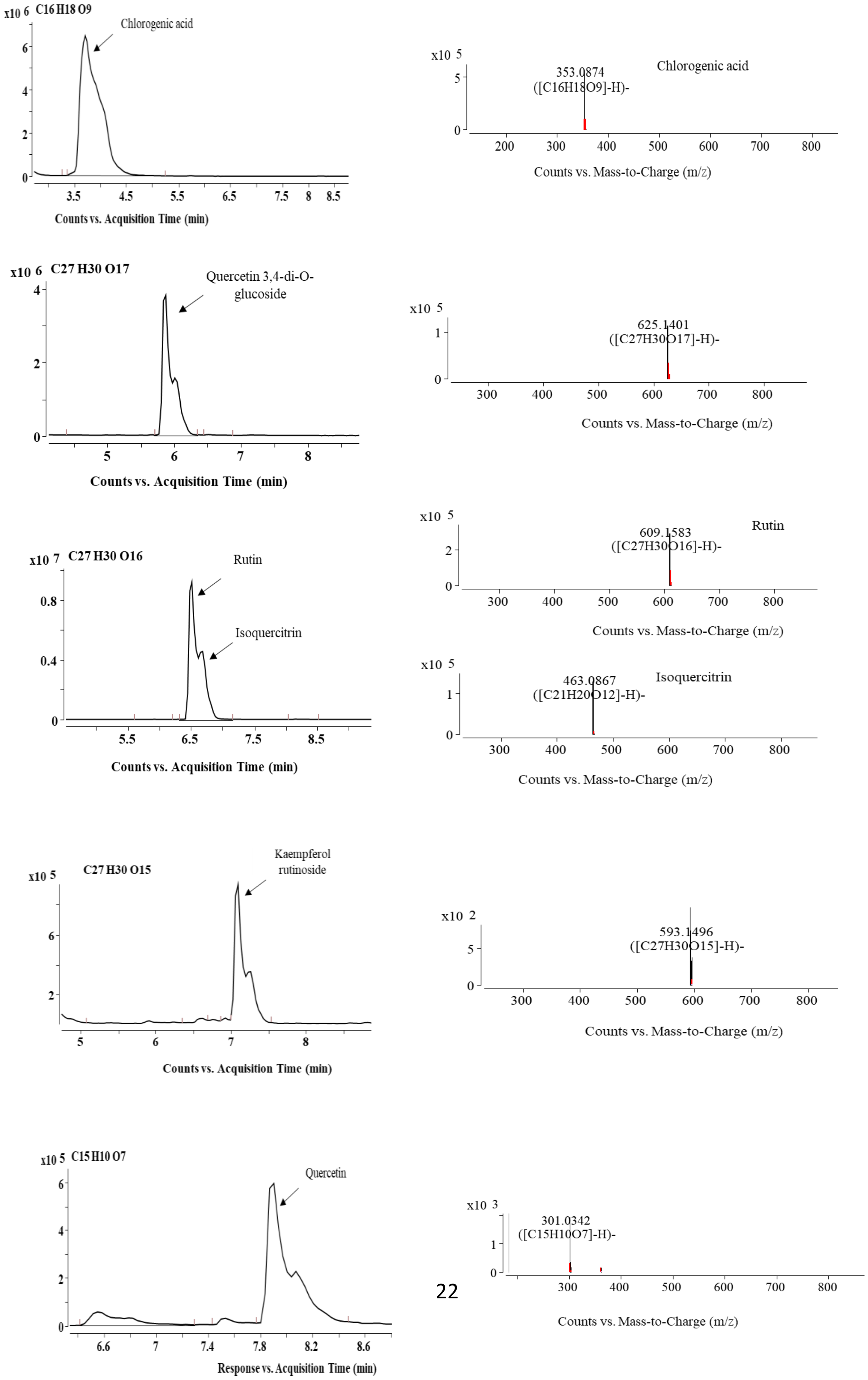

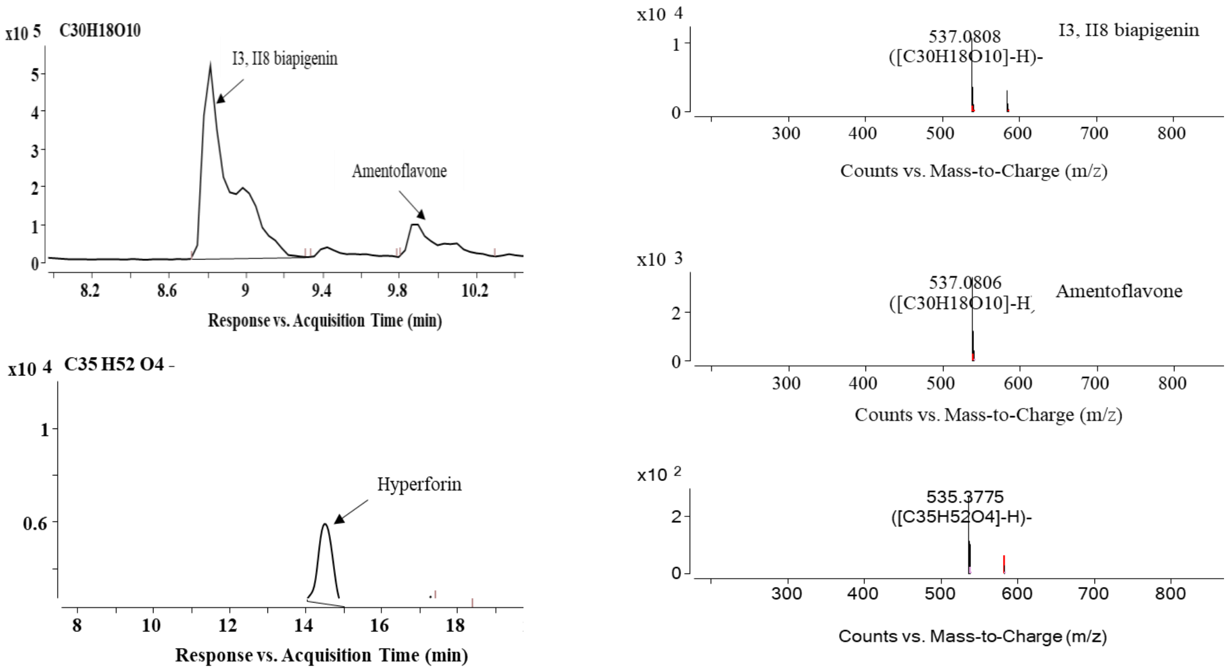
x10 5 C30H18O10
I3, II8 biapigenin
Amentoflavone
Response vs. Acquisition Time (min)
x10 4 C35 H52 O4 -
Hyperforin
Response vs. Acquisition Time (min)
x10 4
537.0808
([C30H18O10]-H)-
I3, II8 biapigenin
Counts vs. Mass-to-Charge (m/z)
x10 3
537.0806
([C30H18O10]-H)
Amentoflavone
Counts vs. Mass-to-Charge (m/z)
x10 2
535.3775
([C35H52O4]-H)-
Counts vs. Mass-to-Charge (m/z)
